# In pursuit of a better broiler: tibial morphology, breaking strength, and ash content in conventional and slower-growing strains of broiler chickens

**DOI:** 10.1016/j.psj.2022.101755

**Published:** 2022-01-30

**Authors:** Midian N. Santos, Tina M. Widowski, Elijah G. Kiarie, Michele T. Guerin, A. Michelle Edwards, Stephanie Torrey

**Affiliations:** ⁎ Department of Animal Biosciences, University of Guelph, Guelph, ON N1G 2W1, Canada; † Campbell Centre for the Study of Animal Welfare, University of Guelph, Guelph, ON N1G 2W1, Canada; ‡ Department of Population Medicine, University of Guelph, Guelph, ON N1G 2W1, Canada; § Ontario Agricultural College, University of Guelph, Guelph, ON N1G 2W1, Canada

**Keywords:** chicken, slow-growth, bone health, genetic, growth rate, lameness

## Abstract

This study was conducted to determine the differences in bone traits in 14 strains of broiler chickens differing in growth rate. The strains encompassed 2 conventional (CONV; ADG_0-48_ >60 g/d) and 12 slower-growing (**SG**) strains classified as FAST (ADG_0-62_ = 53–55 g/d), MOD (ADG_0-62_ = 50–51 g/d), and SLOW (ADG_0-62_ <50 g/d), with 4 strains represented in each SG category. A total of 7,216 mixed-sex birds were equally allocated into 164 pens (44 birds/pen; 30 kg/m^2^) in a randomized incomplete block design, with each strain represented in 8 to 12 pens over 2–3 trials. From each pen, 4 birds (2 males and 2 females) were individually weighed and euthanized at 2 target weights (**TWs**) according to their time to reach approximately 2.1 kg (TW1: 34 d for CONV and 48 d for SG strains) and 3.2 kg (TW2: 48 d for CONV and 62 d for SG strains). Tibiae samples were dissected, and length and diameter were recorded. Left tibiae were used for tibial breaking strength (**TBS**) at both TWs and tibial ash at TW2. At TW1, CONV birds’ tibiae were narrowest and shortest (*P* < 0.001), yet had similar TBS compared to the other categories (*P* > 0.69). At TW2, category (*P* > 0.50) had no effect on tibial diameter, yet CONV birds had the shortest tibiae (*P* < 0.001). The CONV birds had greater TBS:BW ratio than FAST and MOD birds at both TWs 1 and 2 (*P* < 0.039) and similar ash content as the other categories at TW2 (*P* > 0.220). At 48 d of age, CONV birds had the greatest absolute TBS (*P* < 0.003), yet lower TBS:BW ratio than SLOW birds (*P* < 0.001). Tibiae from CONV birds were longer than MOD and SLOW birds, and thicker in diameter than the other categories, yet CONV birds had the lowest dimensions relative to BW (*P* < 0.001) at 48 d, indicating a negative association between accelerated growth and tibial dimensions. These results indicate that differences in functional abilities among categories may be due to differences in morphometric traits rather than differences in bone strength and mineralization.

## INTRODUCTION

Over the past 60 yr, the growth rate of commercial breeds of broiler chickens has increased over 400%, resulting in more efficient birds that need less time to reach market weight (Zuidhof et al., 2014). It has been estimated that 85 to 90% of these improvements are attributed to genetic selection (Havenstein et al., 1994, 2003). However, this improved efficiency comes at a cost, as fast and early growth have been linked to skeletal disorders, leg weakness, and impaired walking ability that can compromise the welfare of the birds (Julian, 1998; SCAHAW, 2000; Meluzzi and Sirri, 2009; Kierończyk et al., 2017).

The possible adverse effects of selection for growth and production traits on leg health and skeletal integrity have been mainly attributed to the structure of the bone (Lilburn, 1994; González-Cerón et al., 2015), suggesting that rapid muscle accretion is not fully accompanied by adequate bone development to support the heavy body weight (**BW**). The imbalance between accelerated growth and skeletal development may lead to a body mass and physical load that is too heavy to be properly supported by immature leg bones at a very early age (Yalcin et al., 2001; Bradshaw et al., 2002; Caplen et al., 2014; González-Cerón et al., 2015; Kierończyk et al., 2017). This imbalance is likely to become greater every year, as the number of days for birds to reach a market weight of approximately 2 kg decreases while the age of skeletal maturity remains the same, at approximately 23 to 27 wk of age (Rath et al., 2000; Sherlock et al., 2010).

Research has shown that fast-growing (**FG**) broiler chickens have an accelerated and early increase in BW compared to a smaller increase in bone development (demonstrated by changes in length and diameter of the femur and tibia over a production cycle of 6 wk) (Applegate and Lilburn, 2002). In addition, FG strains of broiler chickens were reported to have a compromised ability to respond to mechanical load-bearing, suggesting a limited capacity of the skeletal system to adapt to the rapid changes in BW (Pitsillides et al., 1999; Angel, 2007; Rawlinson et al., 2009). However, other studies have suggested that the dimensions of the tibiotarsus of FG strains of broiler chickens are appropriate to provide load support, but the bone itself is fragile, due to high porosity in the cortical bone and low mineral content (Corr et al., 2003; Williams et al., 2004). A recent comparison of morphometric and mechanic traits between a FG and a novel dual-purpose strain (i.e., SG) revealed a similar growth pattern of the tibiotarsus in both strains (Harash et al., 2020), yet differences in tibial weight and dimensions were observed at a similar age and BW. While at a similar age, the SG strain had lighter, shorter and thinner tibiotarsi than FG birds, at a similar BW the tibiotarsus of SG birds were longer, thicker, and heavier than those of FG birds, suggesting SG birds can be reared to market weight without compromising their leg bone integrity and quality (Harash et al., 2020). Other studies have demonstrated the influence of growth rate (Shim et al., 2012b) and genetic strain (Evaris et al., 2019) on several bone traits.

Poor bone quality has been associated with bone deformities, fragility, risk of fractures, and leg weakness (McDevitt et al., 2006; González-Cerón et al., 2015). Bone ash content is a well-validated method to assess bone mineralization (González-Cerón et al., 2015), whereas bone breaking strength is commonly measured to estimate fracture resistance and the force required to bend and break the bone (Kim et al., 2004). In poultry species, breaking strength has been found to be positively correlated with bone weight, ash content, and mineral quality (Shim et al., 2012b). These parameters, along with anatomical measurements (e.g., length, diameter, area, angulation) are commonly used to assess bone quality, development, and morphology (Shim et al., 2012b; Toscano et al., 2013; González-Cerón et al., 2015). However, there is some inconsistency in the literature regarding the relationship between bone traits and walking ability in broiler chickens.

The possible negative impacts of selection for early and accelerated growth on bone health and walking ability may occur because the emphasis on productive traits results in less energy allocated to other metabolic processes (Tallentire et al., 2016). Furthermore, increased locomotor activity has been shown to improve walking ability and bone development (Reiter and Bessei, 1995; Pedersen et al., 2020). Therefore, the low activity levels consistently reported in FG birds (Bizeray et al., 2000; Bokkers and Koene, 2003; Dixon, 2020) may exacerbate the incidence of some leg disorders that cause lameness. However, in addition to the rapid increase in BW, other factors can induce skeletal problems in broiler chickens, including nutrition deficiencies, infectious diseases, mechanical trauma, and the interaction of these factors (Angel, 2007; Kierończyk et al., 2017). Furthermore, bone health and skeletal integrity have been included in breeding programs, resulting in a reduction of bone disorders that were commonly found in broiler chickens decades ago (Angel, 2007; Whitehead, 2007). Indeed, the percentage of birds with tibial dyschondroplasia (**TD**) and other growth plate deformities that can impact walking ability has decreased since the 1980s (Veltmann and Jensen, 1980,^1981^; McKay et al., 2000). These improvements can be attributed to better nutrition, management practices, and development of genetic strategies incorporating leg health and robustness (McKay et al., 2000; Whitehead, 2007). However, despite these improvements, it has been estimated that 14 to 30% of broiler chickens raised worldwide have poor gait score (Sanotra et al., 2003; Bassler et al., 2013; Kittelsen et al., 2017; Vasdal et al., 2018), which indicates that impaired walking ability is still an ongoing problem in broiler production.

Because there is a lack of fully effective strategies to improve leg health without influencing growth, there is an increasing interest in the use of slower-growing (**SG**) strains to decrease skeletal abnormalities and improve the walking ability and welfare of broiler chickens (Bessei, 2006; Shim et al., 2012b; Dixon, 2020). Although comparisons between a few strains of FG and SG broilers have been performed (Bokkers and Koene, 2003; Dixon, 2020; Mancinelli et al., 2020), there is a scarcity of studies that investigate different strains of broiler chickens raised under similar conditions and tested at a similar BW. In addition, although the term “fast-growing” commonly refers to conventional broiler chickens that are intensively selected for meat production and reach market weight at an early age (about 2.5 kg in 40 d; ADG ≥60 g/d), the term "slow-growing" encompasses a wide range of growth rates, representing a heterogenous group of birds, commonly raised in alternative production systems (Doğan et al., 2019; Mancinelli et al., 2020). In this context, bone quality and morphologic traits were investigated in 14 strains of broiler chickens (2 FG and 12 SG), encompassing a wide range of growth rates. It was hypothesized that SG strains of broiler chickens would have better bone quality than FG strains, indicated by greater bone breaking strength and ash content.

## MATERIALS AND METHODS

### Hatching and Husbandry

The procedures carried in this study were reviewed and approved by the University of Guelph's Animal Care Committee (AUP #3746) and were in accordance with the Canadian Council for Animal Care's guidelines (2009).

This study is part of a multidisciplinary project conducted to assess production performance, meat quality, behavior, physiology, leg health, inactivity, and welfare of FG and SG strains selected for distinct growth rates, described in other associated papers (Mohammadigheisar et al., 2020, 2021; Dawson et al., 2021; Torrey et al., 2021; Santos et al., 2021). The complete details regarding incubation conditions, animal handling, husbandry, management, and housing are available elsewhere (Torrey et al., 2021).

Briefly, a total of 8 trials were conducted at the Arkell Poultry Research Station (Guelph, ON, Canada), with each trial representing a typical broiler production cycle, from incubation and hatch to slaughter. A total of 5 to 7 strains were tested in each trial. Fertile eggs from 14 strains (2 FG and 12 SG) were incubated simultaneously in each trial under similar and standardized conditions at the federally inspected facility at Arkell Poultry Research Station. A total of 7,216 birds were reared over 8 trials in a single room, with 28 floor pens (160 cm × 238 cm; width × length) and expected stocking density of 30 kg/m^2^. In total, 164 groups of birds were reared into the 28 identical pens over a 2-yr period. For details about experimental design and rearing conditions refer to Torrey et al. (2021).

According to the location of the pens, the room was divided into four blocks due to micro-climate differences at a pen level detected in pilot studies. Overall, each strain was tested in up to 3 trials, with a total of four pens per trial representing each block of the room, totaling 12 pens per strain, except for strains G and M due to the availability of fertile eggs for these strains (Torrey et al., 2021).

Birds from each strain were sexed at hatch, equally divided, and allocated into the experimental pens, with a total 44 birds per pen (22 males and 22 females). In each trial the group weight of each pen was obtained to maintain a similar initial BW for each strain at placement. A total of 12 birds per pen (6 males and 6 females), were randomly selected and used as focal birds. These birds were individually weighed, wing banded, and identified using a livestock paint. The focal birds were used to assess several parameters (i.e., behavior, health, meat quality, and physiology) described in other associated studies (Dawson et al., 2021; Santos et al., 2021, 2022). All the birds were vaccinated against infectious bronchitis, coccidiosis, and Marek's disease (Torrey et al., 2021).

An all vegetarian, antibiotic-free feed formulated for slower growth was fed ad libitum for all the birds. Light intensity was kept at 20 lux, with 23 h of light (**L**) and 1 h of dark (**D**) on the first 3 d to allow chicks to allocate resources in the pen. Subsequently, a 16L:8D regime was used, with a continuous dark period. Temperature was kept at 32°C at placement and gradually decreased to 21°C at 5 wk of age.

To determine the impacts of BW on the variables evaluated in the study, strains were processed at 2 target weights (**TWs**) based on their expected time to reach 2.1 kg (**TW 1**) and 3.2 kg (**TW 2**). Fast-growing birds were processed at 34 d and 48 d, which corresponds to TW 1 and TW 2, respectively while SG strains were processed two weeks later at 48 d and 62 d. These days were selected as an attempt to compare the birds at a similar BW (2.1 and 3.2 kg) and similar age (48 d).

### Tibial Morphology Parameters

A total of 4 wing-tagged focal birds (2 males and 2 females) from each pen were individually weighed and labeled for identification purposes the day before processing, at either TW 1 or TW 2. These birds were selected previously to determine birds’ mobility as measured by latency-to-lie and group obstacle tests as described in an associated study (Santos et al., 2022). The group weight from each pen to be processed was obtained to determine the final BW and production performance of each strain (Torrey et al., 2021). Feed was removed from each pen the night before processing at 23:00. Birds had free access to water until loading. The next morning, the 4 focal birds were killed by cervical dislocation, left to cool at 4°C for a maximum of 6 h, and then kept in a freezer at −20°C until analyses. Prior to dissections, carcasses were transferred to a cooler room at 4°C and thawed for 48 to72 h depending on body size to facilitate dissections and separation of tissues. Dissections were performed by three trained researchers to keep measurements consistent across strains.

Both left and right tibiae were dissected and completely defleshed to remove adherent soft tissues. The length of each tibia was measured from the lateral intercondylar tubercle to the inferior articular surface and the diameter was measured at the midpoint of the diaphysis using a digital caliper (Fisher Scientific carbon fiber composite digital calipers; Toronto, ON, Canada; Resolution: 0.1 mm, Accuracy: ± 0.2 mm). The mean length and diameter from both tibiae were measured and used as a single value for each variable.

### Tibial Breaking Strength

After morphometric measurements, the bone samples were placed in labeled plastic bags and stored in a freezer at −20°C. The left tibiae from the focal birds were thawed for 24 h in a cooler at 4°C prior to breaking. To measure tibial breaking strength (**TBS**), a 3-point bending Instron material tester with Bluehill Universal software (Model Material Testing, Norwood, MA) was used. Each bone sample was placed in the same orientation and held by cradle support with a span of 5 cm. A 5kN load cell at a speed of 20 mm/s was applied at the midpoint of the bone, with a fixed distance of 50 mm between upper and lower anvils. Details about speed and methodology used are provided in the supplementary material. The maximum force required to break the bone was detected from the deformation curve and was used to determine the breaking strength in Newtons (**N**). The absolute values along with TBS expressed as a proportion to the BW were determined.

### Tibial Dyschondroplasia and Tibial Composition

Following breaking strength, the left tibiae from the focal birds were stored in a freezer at −20°C until subject to the determination of TD and tibial composition. Due to time constraints and logistics, only bones from birds killed at TW 2 were used for these analyses. The tibia from each bird was thawed for 1 h at room temperature. The thawed weight was recorded using an analytical scale (Mettler AC 88 digital balance; Mississauga, ON, Canada; Accuracy: 0.0001 g). The proximal end of the left tibia was cut longitudinally to determine the presence of TD as described by Shim et al., 2012a and all the pieces were kept for the determination of tibial composition.

Subsequently, each tibia was placed in hexane for 2 d for fat extraction (Kiarie et al., 2015) and later the defatted tibiae were transferred to pre-weighed crucibles and dried in an oven for 24 h at 105°C. Tibial dry matter weight (the remaining content after removal of fat and moisture) was obtained using the same analytical scale as above. Next, crucibles and dry tibiae were transferred to a muffle furnace for 12 h at 600°C as described by Khanal et al. (2019). The samples were placed in a desiccator until they reached room temperature and the final weight was recorded to determine ash content relative to the dry weight of the tibia. The total organic matter content of the tibia was determined by subtracting ash content from the dry matter content. The organic matter weight was divided by the ash weight to estimate the ratio of organic to inorganic matter and both ash and organic matter were expressed as absolute values, while ash weight was also expressed relative to the tibial length as described by McDevitt et al. (2006). To provide a quantitative assessment of tibial weights considering the differences in BW, tibial dry weight, and ash content were also expressed relative to the BW (Shim et al., 2012b; Guo et al., 2019).

### Statistical Analyses

To facilitate analyses, strains were grouped into 4 categories based on their similar growth rates to TW 2 (48 d for FG and 62 d for SG strains, respectively). The strains were categorized as conventional (**CONV**; strains B and C; ADG = 66.0–68.7 g/d), fastest slow-growing (**FAST**; strains F, G, I, and M; ADG_0-62_ = 53.5–55.5 g/d), moderate slow-growing (**MOD**; strains E, H, O, and S; ADG_0-62_ = 50.2–51.2 g/d), and slowest slow-growing (**SLOW**; strains D, J, K, and N; ADG_0-62_ = 43.6–47.7 g/d). Comparisons between categories and within categories were assessed for each dependent variable. Comparisons between categories were conducted to assess differences at different growth rates, whereas comparisons within categories were conducted to assess differences among strains at a similar growth rate.

Data were analyzed as an incomplete block design using a Generalized linear mixed model (**GLIMMIX**) in SAS, version 9.4 (SAS Institute Inc., Cary, NC), with pen as the experimental unit. Different models were used to evaluate the effects of TW and age on a number of dependent variables. The *TW model* was used to assess tibial morphology (length and diameter) and TBS when the birds were evaluated at a similar BW. In these models, category, strain nested within category, sex, TW, and their interactions were included as main effects. The interactions in the TW models included category × TW, category × sex, category × sex × TW, strain (category) × TW, strain (category) × sex, strain (category) × sex × TW. These models allowed comparisons at the 2 TWs to investigate the effects of BW on tibial characteristics for both FG and SG strains. The *age model* was used to assess differences in tibial morphology traits when both FG and SG birds were 48 d (TW 1 and TW 2 for SG and FG birds, respectively). In these models, category, strain nested within category, and sex were included as main effects. The interactions between category × sex, and strain (category) × sex were kept in the model if significant. Because tibial dry matter, ash, and organic content were only evaluated in birds processed at TW 2, another model was used, named *TW 2 model*, which omitted the TW and its interactions. In these models, category, strain (category), and sex were included as main effects, with the inclusion of the interactions between category × sex and strain (category) × sex, which were kept in the model if significant. The random effects for all models included trial (i.e., production cycle) and block nested within the trial.

Differences between categories and among strains within each category were compared using contrast statements. For all models, the residuals were checked for normality using a quantile-quantile plot and Shapiro-Wilk test. Linearity, randomness, and homogeneity of residuals were assessed using scatterplots and boxplots of studentized residuals. Residual analysis was used to select the most appropriate distribution that met all of the model assumptions, with Gaussian distribution used by default for those variables that met all the assumptions. For BW, tibial dimensions traits (absolute and relative to the BW), and TBS (absolute and relative to the BW), a lognormal distribution was required to meet the model assumptions. Differences were considered significant at adjusted *P* < 0.05. Pairwise comparisons were corrected using Tukey adjustment to explore multiple comparisons and differences between categories, strains, and sex. Differences between sex or TW are not provided in data tables, though they are described in the Results section with their respective *P*-value, if significant.

## RESULTS

Differences in tibial parameters at both TWs are described as differences between categories of strains (Table 1) and between strains within the same category (Supplementary Tables 1–4). As expected, category affected most of the variables evaluated at both TWs. For variables measured at the same age (i.e., BW, tibial breaking strength, length, and diameter), only differences among categories are provided in Table 2, as birds from strains within the same category were evaluated at the same age. Significant interactions between category, TW, and sex are not presented here but are included in the Supplementary Table 5. Differences between TWs and sexes are described in the text if significant.

**Table 1:** Effect of category on body weight (BW), tibial breaking strength (TBS), tibial morphology, and tibial ash and organic content (LS-means ± SEM) at Target Weights 1 and 2. At Target weight 1, birds of conventional and slower-growing strains were 34 and 48 d of age, respectively. At Target Weight 2, birds of conventional and slower-growing strains were 48 and 62 d, respectively.

	Category			
Variable				
Target weight 1^1^	CONV	FAST	MOD	SLOW
BW (g)	1,857 ± 40.9^d^	2,519 ± 38.6^a^	2,359 ± 34.2^b^	2,015 ± 29.5^c^
TBS (N)^2^	290.1 ± 12.55	300.3 ± 8.87	284.2 ± 7.95	274.1 ± 7.60
TBS:BW (N/kg)^3^	156.0 ± 6.49^a^	119.0 ± 3.42^c^	120.7 ± 3.26^c^	136.0 ± 3.63^b^
Diameter (mm)	7.21 ± 0.144^c^	8.59 ± 0.116^a^	8.33 ± 0.108^a^^b^	8.18 ± 0.105^b^
Length (mm)	95.6 ± 0.75^c^	116.1 ± 0.61^a^	112.5 ± 0.56^b^	111.8 ± 0.55^b^
Diameter:BW (mm/kg)	3.87 ± 0.081^a^	3.40 ± 0.047^b^	3.54 ± 0.047^b^	4.04 ± 0.053^a^
Length:BW (mm/kg)	51.48 ± 0.954^b^	46.10 ± 0.583^c^	47.70 ± 0.572^c^	55.50 ± 0.657^a^
Length:Diameter	13.28 ± 0.226	13.50 ± 0.154	13.50 ± 0.147	13.66 ± 0.148
Target Weight 2^4^				
BW (g)	3,264 ± 60.0^a^^b^	3,437 ± 48.5^a^	3,185 ± 42.3^b^	2,844 ± 37.6^c^
TBS (N)	362.8 ± 12.63^a^	339.4 ± 9.30^a^^b^	318.6 ± 8.13^b^	320.6 ± 8.08^b^
TBS:BW (N/kg)	111.2 ± 3.42^a^	98.7 ± 2.60^a^	100.0 ± 2.46^b^	112.7 ± 2.74^a^
Diameter (mm)	9.05 ± 0.143	9.31 ± 0.012	9.29 ± 0.109	9.26 ± 0.108
Length (mm)	116.9 ± 0.73^c^	129.9 ± 0.64^a^	128.3 ± 0.59^a^^b^	127.6 ± 0.58^b^
Diameter:BW (mm/kg)	2.78 ± 0.046^b^^c^	2.71 ± 0.036^c^	2.90 ± 0.035^b^	3.25 ± 0.039^a^
Length:BW (mm/kg)	35.80 ± 0.528^d^	37.80 ± 0.444^c^	40.28 ± 0.439^b^	44.90 ± 0.489^a^
Length:Diameter	12.90 ± 0.173^b^	13.95 ± 0.149^a^	13.81 ± 0.137^a^	13.77 ± 0.136^a^
Dry matter wt (g)^5^	9.98 ± 0.207^c^	11.62 ± 0.154^a^	10.85 ± 0.161^b^	10.39 ± 0.163^b^^c^^c^
Ash wt (g)	3.95 ± 0.091^c^	4.61 ± 0.074^a^	4.30 ± 0.073^b^	4.18 ± 0.068^b^^c^
Organic matter wt (g)	6.04 ± 0.125^c^	7.01 ± 0.910^a^	6.54 ± 0.102^b^	6.21 ± 0.105^b^^c^
Dry matter wt:BW (%)	3.05 ± 0.046^c^	3.38 ± 0.030^b^	3.40 ± 0.043^b^	3.65 ± 0.042^a^
Ash wt:BW (%)	1.21 ± 0.015^c^	1.33 ± 0.014^b^	1.35 ± 0.018^b^	1.47 ± 0.017^a^
Ash content (%)	39.42 ± 0.363	39.62 ± 0.278	39.63 ± 0.326	40.35 ± 0.323
Organic matter (%)	60.58 ± 0.363	60.38 ± 0.278	60.37 ± 0.326	59.65 ± 0.323
Organic:Inorganic	1.53 ± 0.024	1.52 ± 0.017	1.52 ± 0.023	1.49 ± 0.019
Ash:Length (g/mm)	0.34 ± 0.006^a^^b^	0.35 ± 0.005^a^	0.33 ± 0.005^b^	0.33 ± 0.005^b^

1 Number of birds per category at Target Weight 1: CONV: n = 34; FAST: n = 78; MOD: n = 85; SLOW: n = 86.

2 Absolute tibial breaking strength (TBS). Maximum TBS expressed in newtons (N).

3 Relative tibial breaking strength (TBS). Maximum TBS was obtained in newtons (N) and adjusted for the BW.

4 Number of birds per category at Target Weight 2: CONV: n = 54; FAST: n = 95; MOD: n = 101; SLOW: n = 103.

5 Tibial dry matter, organic and inorganic content were only obtained at Target Weight 2.

a-d Different superscripts within the same row represent significant differences between categories (*P* < 0.05).

**Table 2:** Differences in body weight (BW), tibial breaking strength (TBS) and tibial morphology (LS-means ± SEM) among categories at the same age (48 d).

	Category^1^			
Variable	CONV	FAST	MOD	SLOW
BW (g)	3,257 ± 56.4^a^	2,518 ± 32.8^b^	2,359 ± 32.8^c^	2,015 ± 27.9^d^
TBS (N)^2^	363.7 ± 13.60^a^	305.9 ± 9.54^b^	285.0 ± 8.51^b^^c^	271.0 ± 8.02^c^
TBS:BW (N/kg)^3^	111.8 ± 4.03^b^	121.0 ± 3.66^a^^b^	121.8 ± 3.48^a^^b^	134.1 ± 3.83^a^
Diameter (mm)	9.07 ± 0.139^a^	8.59 ± 0.111^b^	8.33 ± 0.104^b^^c^	8.18 ± 0.101^c^
Length (mm)	117.1 ± 0.71^a^	116.1 ± 0.60^a^	112.4 ± 0.55^b^	111.8 ± 0.55^b^
Diameter:BW (mm/kg)	2.78 ± 0.039^c^	3.39 ± 0.040^b^	3.53 ± 0.040^b^	4.03 ± 0.046^a^
Length:BW (mm/kg)	35.95 ± 0.496^c^	46.00 ± 0.548^b^	47.70 ± 0.537^b^	55.40 ± 0.617^a^
Length:Diameter	12.88 ± 0.167^b^	13.50 ± 0.150^a^	13.50 ± 0.142^a^	13.66 ± 0.143^a^

1 Number of birds per category at 48 d of age: CONV: n = 54; FAST: n = 78; MOD: n = 85; SLOW: n = 86.

2 Absolute tibial breaking strength (TBS). Maximum TBS expressed in newtons (N).

3 Relative tibial breaking strength (TBS). Maximum TBS was obtained in newtons (N) and adjusted for the BW.

a-d Different superscripts within the same row represent significant differences between categories (*P* < 0.05).

### Body Weight and Leg Traits at Target Weight 1 and 2

#### BW

The BW was affected by category, TW, strain, and sex (*P* < 0.001). However, category interacted with TW (*P* < 0.001), indicating that the effect of category on BW depended on the TW (Table 1). At TW 1, a significant difference was observed among the categories with CONV birds having the lowest BW, followed by SLOW, MOD, and FAST birds. However, at TW 2, SLOW birds had the lightest BW, while CONV birds were similar to FAST and MOD birds. As expected, males were heavier than females (2,901 ± 19.2 g vs. 2,376 ± 16.7 g).

#### Tibial Breaking Strength

Overall, there was an increase in absolute TBS from TW 1 to TW 2 (*P* < 0.001; 286.9 ± 4.70 N vs*.* 334.9 ± 4.79 N). However, differences in TBS among categories varied according to the TW. At TW 1, category did not affect TBS, whereas at TW 2, CONV birds had greater TBS than MOD and SLOW birds (Table 1; *P* < 0.022). Sex influenced TBS, with males being greater than females (*P* < 0.001; 353.8 ± 4.87 N vs. 271.7 ± 3.96 N), with no interaction between sex and category or sex and strain (*P* > 0.083).

#### Tibial Diameter

Tibial diameter differed among TWs, categories, strain, and sex (*P* < 0.001). Overall, tibiae became wider in diameter from TW 1 to TW 2 (8.06 ± 0.061 mm vs. 9.23 ± 0.061 mm). However, a TW by category interaction was found (Table 1; *P* < 0.001). At TW 1, CONV birds had the smallest tibial diameter while at TW 2, no difference in tibial diameter was observed among the categories. Despite the significant differences among strains (*P* < 0.001), strain within category did not influence tibial diameter (*P* > 0.068 for all combinations among strains within category at both TWs). Males had wider tibial diameter than females (*P* < 0.001; 9.31 ± 0.054 mm vs. 7.98 ± 0.048 mm), with no significant interaction between category or strain with sex and TW (*P* > 0.204).

#### Tibial Length

Tibial length was affected by TW, category, strain, and sex (*P* < 0.001). Similar to diameter, tibial length increased from TW 1 to TW 2 (*P* < 0.001; 108.7 ± 0.319 mm vs. 125.6 ± 0.321 mm). At both TWs, CONV had shorter tibiae than the other categories. Within categories, CONV and FAST birds had different tibial lengths at TW 1 and TW 2, respectively. Among CONV strains, at TW 1, birds from strain B had longer tibiae than strain C (Supplementary Table 1), whereas among FAST strains, at TW 2, strain I had longer tibiae than strain F (Supplementary Table 2). Overall, males had longer tibiae than females (P < 0.001; 120.4 ± 0.297 mm vs*.* 113.4 ± 0.291 mm). However, at TW 1, sex did not influence tibial length for CONV birds, whereas at TW 2, sex influenced tibial length in all categories, with males having longer tibiae than females (category × TW × sex interaction; *P* = 0.009, Supplementary Table 5).

#### Relative Tibial Breaking Strength

Overall, relative TBS decreased as the birds grew (*P* < 0.001; TW 1= 131.8 ± 2.09 N/kg, TW 2 = 105.4 ± 1.46 N/kg). Category affected relative TBS per unit of BW at both TWs (Table 1; *P* = 0.038). At TW 1, CONV birds had the highest relative TBS, while at TW 2, CONV and SLOW birds had similar relative TBS that was higher than FAST and MOD birds. At TW 1, no difference among strains within category was observed. At TW 2 among MOD strains, strain H had higher relative TBS than strain O (Supplementary Table 3; *P* = 0.013) while among SLOW strains, strain D had higher relative TBS than J (Supplementary Table 4; *P* = 0.006). Males had higher relative TBS than females (*P* < 0.001; 121.5 ± 1.61 N/kg, *vs.* 114.4 ±1.61 N/kg), with no interaction between sex, category, and TW or sex, strain, and TW (*P* > 0.170)

#### Relative Tibial Diameter

Birds had greater relative tibial diameter at TW 1 than those at TW 2 (*P* < 0.001; 3.69 ± 0.029 mm/kg *vs.* 2.91 ± 0.019 mm/kg). However, there was a category by TW interaction that affected tibial diameter per unit of BW (Table 1; *P* < 0.001). At TW 1, CONV and SLOW birds had similar relative tibial diameter, which was greater than FAST and MOD birds. At TW 2, CONV, FAST, and MOD birds had lower relative tibial diameter than SLOW birds. Overall, males had lower relative tibial diameter than females (*P* < 0.001; 3.19 ± 0.020 mm/kg vs*.* 3.36 ± 0.022 mm/kg). The interaction between sex, category, and TW was not significant (*P* = 0.682).

#### Relative Tibial Length

Overall, relative tibial length was greater in TW 1 compared to TW 2 (*P* < 0.001; 49.84 ± 0.352 mm/kg vs. 39.53 ± 0.241 mm/kg). However, TW interacted with category (Table 1; *P* < 0.001). At TW 1, SLOW birds had the greatest length relative to the BW, while CONV birds were greater than FAST and MOD birds. At TW 2, relative length decreased as the growth rate increased among categories (CONV < FAST < MOD < SLOW). Males had lower relative tibial length than females (*P* < 0.001; 41.29 ± 0.229 mm/kg vs*.* 47.73 ± 0.276 mm/kg).

#### Ratio of Tibial Length to Diameter

Length:diameter was not affected by TW (*P* = 0.172). However, the ratio of length:diameter was affected by category, strain, and sex (*P* < 0.002). At TW 1, category did not affect length:diameter, whereas at TW 2, CONV birds had a lower ratio than the other categories (Table 1; *P* < 0.001). Within categories, FAST and SLOW strains differed in length:diameter at TW 1. Among the FAST strains, strain F had lower length:diameter than strains G (*P* = 0.049) and M (*P* = 0.009) (Supplementary Table 2), while among SLOW strains, strain D had higher ratio than strain N (*P* = 0.046; Supplementary Table 4). However, these differences were not found at TW 2. Overall, males had lower tibial length:diameter than females (*P* < 0.001; 12.92 ± 0.067 mm/kg vs. 14.18 ± 0.076 mm/kg).

### Tibial Content – Dry Matter, Organic, and Inorganic Content at TW 2

Tibial dry matter weight differed by category and sex (Table 1; P < 0.001). The CONV birds had the lightest tibial dry matter, which was similar to SLOW and lighter than both FAST and MOD birds. Overall, males had heavier tibial dry matter than females (P < 0.001; 12.35 ± 0.104 g vs*.* 9.07 ± 0.118 g). However, there was an interaction between sex and category (*P* < 0.001; Supplementary Table 5). Dry matter weight was not affected by category among females, whereas among males, FAST birds had heavier dry matter weight than the other categories.

Both tibial ash weight and the weight of organic content followed a similar pattern observed for tibial dry matter, with CONV being similar to SLOW birds yet lighter than both FAST and MOD birds (Table 1). While males had heavier tibial ash than females (*P* < 0.001; 4.94 ± 0.046 g vs. 3.57 ± 0.053 g), an interaction between sex and category affected tibial ash weight (*P* < 0.001, Supplementary Table 5). Similar to the interaction observed for tibial dry matter weight, no difference in tibial ash weight was observed among categories for females, while for males there was an effect of category, with CONV birds being lower than the other categories. Males had heavier organic matter weight than females (*P* < 0.001; 7.40 ± 0.066 g vs. 5.49 ± 0.076 g), with an interaction between sex and category (*P* = 0.009) that followed a similar pattern to those observed for tibial dry matter and ash weight (Supplementary Table 5).

Category, strain, and sex affected tibial dry matter and ash weight relative to the BW (Table 1; *P* < 0.001). Among categories, CONV birds had the lowest and SLOW birds had the highest values, while FAST and MOD showed intermediate values. Within categories, only FAST strains differed in dry matter weight relative to BW (Supplementary Table 2; *P* < 0.001), with strain F being lower than strain M (*P* = 0.041). For tibial ash weight relative to the BW, significant differences were detected in FAST and MOD strains. Among FAST strains (Supplementary Table 2), strain F had lower tibial ash weight per unit of BW than strains I (*P* = 0.006) and M (*P* = 0.001). Among MOD birds (Supplementary Table 3), strain E had higher tibial ash weight relative to BW than strain O (*P* = 0.041). Males had greater tibial dry matter relative to the BW than females (*P* <0.001; 3.49 ± 0.026 g/kg vs. 3.18 ± 0.029 g/kg,) and there was no interaction between sex and category or sex and strain (*P* ˃ 0.200). Overall, males had higher tibial ash weight per unit of BW than females (*P* < 0.001; 1.39 ± 0.010 g/kg vs. 1.25 ± 0.012 g/kg). However, sex interacted with category (*P* = 0.033; Supplementary Table 5); there was an effect of sex in all the categories except CONV, in which females and males had similar tibial ash weight relative to BW.

Tibial ash content did not differ among categories (*P* = 0.354), but it was affected by strain (*P* < 0.001) and sex (P = 0.038). Within categories, only FAST strains differed in tibial ash content (Supplementary Table 2). Strain F had lower tibial ash content than strain I (*P* < 0.001). Males had greater tibial ash content than females (40.08 ± 0.209% vs. 39.43 ± 0.241%) and no interaction between sex and category or sex and strain was found (*P* ˃ 0.620).

While category had no effect on organic matter content and organic:inorganic matter (*P* > 0.354), these variables were affected by strain and sex (*P* < 0.037). In the FAST category (Supplementary Table 2), strain F had greater organic matter content (*P* < 0.001) and organic:inorganic (*P* = 0.001) than strain I. Males had lower tibial organic content (*P* = 0.038; 59.91 ± 0.209% vs. 60.56 ± 0.241%) and organic relative to inorganic matter (*P* = 0.037; 1.50 ± 0.014 vs. 1.55 ± 0.016) than females. There was no interaction between sex and category or sex and strain for both tibial organic matter content and organic relative to inorganic matter (*P* ˃ 0.583).

The amount of ash per unit of length of the tibial [ash: length (g/mm)] was influenced by category and sex (*P* < 0.001). Among categories, CONV birds did not differ from the remaining categories, while FAST was greater than MOD and SLOW birds (Table 1). Overall, males had higher tibial ash:length than females (*P* < 0.001; 0.379 ± 0.003 g/mm vs. 0.295 ± 0.004 g/mm). However, among females, category did not affect tibial ash:length, whereas among males, FAST was greater than CONV and SLOW males, resulting in an interaction between category and sex (*P* = 0.012, Supplementary Table 5).

### Tibial Dyschondroplasia at TW 2

Due to the low number of birds affected by TD, statistical analyses were not performed. Therefore, only descriptive statistics are included for this trait. Based on macroscopic examination of the growth plate of the focal birds, the overall incidence of TD was 2.58%, with FAST and MOD birds accounting for 77.78 % of the TD observed in our study. While TD was not found in CONV birds, FAST, MOD, and SLOW birds exhibited incidences of TD at 1.09, 6.06, and 1.96%, respectively. The MOD birds had a TD incidence over twice as high as the overall incidence of TD with strains O and S exhibiting incidences of 8.70 and 17.39%, respectively. The other strains that had TD were: FAST, strain I (4.35%) and SLOW, strains J (4.00%) and K (4.17%). The other strains had no evidence of TD in any samples. All the birds affected only showed mild TD lesions.

### Body Weight and Leg Traits at a Similar Age

Differences in BW and leg traits obtained at the same age (48 d) among categories are provided in Table 2. Category (*P* < 0.006) and sex (*P* < 0.034) affected all variables evaluated at the same age. Because the interactions between sex and category were not significant for any trait (*P* ˃ 0.05), only the main effects of category and sex are presented.

As expected, BW was greater as the growth rate was higher among categories (*P* < 0.001; CONV ˃ FAST ˃ MOD ˃ SLOW) and males were heavier than females (*P* < 0.001; 2,754 ± 24.1 g, vs. 2,267 ± 20.5 g). At 48 d of age, CONV birds had the highest TBS among the categories (*P* < 0.002). Sex had an effect within all categories, with males exhibiting greater TBS than females (*P* < 0.001; 344.6 ± 7.06 N vs. 269.0 ± 5.81 N). When TBS was expressed per unit of BW, CONV was lower than SLOW (*P* < 0.001) birds, while FAST and MOD birds were similar and not different from CONV and SLOW birds. In all categories, males had higher TBS:BW than females (*P* = 0.034; 124.9 ± 2.43 N/kg vs. 118.4 ± 2.42 N/kg,).

Among categories, CONV birds had wider tibiae than the other categories (*P* < 0.001). Males had wider tibiae than females (*P* < 0.001; 9.15 ± 0.073 mm vs. 7.96 ± 0.066 mm). Tibial length differed by category (*P* < 0.001), with CONV and FAST birds being similar and longer than MOD and SLOW birds (*P* < 0.001). As expected, males had longer tibiae than females (*P* < 0.001; 117.4 ± 0.379 mm vs. 111.3 ± 0.373 mm).

Tibial diameter and length per unit of BW (mm/kg) followed a similar pattern, with CONV birds being lower than the other categories (*P* < 0.001) and FAST and MOD birds being similar, yet lower than SLOW birds (*P* < 0.001). Males had lower relative tibial diameter (*P* < 0.001; 3.32 ± 0.026 mm/kg vs*.* 3.50 ± 0.029 mm/kg) and relative length (*P* < 0.001; 42.43 ± 0.308 mm/kg vs. 49.00 ± 0.370 mm/kg) than females, which was consistent in all categories.

The CONV birds had the lowest tibial length:diameter compared to the other categories (*P* < 0.031). Males had lower tibial length relative to diameter than females (*P* < 0.001; 12.80 ± 0.091 vs. 13.97 ± 0.103), which was observed in all categories.

## DISCUSSION

The link between selection for accelerated growth and susceptibility to leg disorders in broiler chickens has been well-documented (Julian, 1998; Williams et al., 2004; Shim et al., 2012b; Dixon, 2020). Since reducing the growth rate decreases leg disorders to some extent, it has been suggested that the use of SG strains may decrease leg abnormalities that cause both welfare and economic issues in the poultry industry (Julian, 1998; Bessei, 2006; Shim et al., 2012a,b). Therefore, the aim of this study was to investigate the differences in tibial morphology, breaking strength, and composition (inorganic and organic content) as indicators of bone quality and bone measurements in 14 strains of broiler chickens (separated into 4 categories based on similarity of growth rate to TW 2) raised under similar conditions. Category affected most of the variables evaluated at TW 1 and TW 2, whereas only a few differences were found between strains within category, suggesting similar selection criteria for bone quality and morphology in strains selected for similar growth rates.

### Effect of Category

#### BW

Even though skeletal disorders are multifactorial conditions, growth rate and BW are among the factors considered to play a crucial role in the incidence of leg abnormalities (Julian, 1998; Bessei, 2006; Shim et al., 2012b). In addition, bone development and morphology are affected by age (Lilburn, 1994; Talaty et al., 2009). To account for the effect of growth rate, BW, and age, FG and SG strains of broiler chickens representing a wide range of growth rates were evaluated at a similar TW and age. However, because the ADG ranged from 43.6 to 68.7 g/d, differences in BW were observed among the categories (Santos et al., 2021). When possible, traits were adjusted relative to BW to account for the differences.

#### Tibial Breaking Strength

Bone breaking strength is a measure of the toughness and capacity of the bone to endure stress and resist fracture (Rath et al., 2000). This variable is affected by different properties, including shape, size, mineral and organic matrices, and collagen crosslinks (Rath et al., 2000; Turner, 2006; Foutz et al., 2007). Bone breaking strength has been shown to be correlated with cortical bone thickness, a crucial indicator of bone development and quality (Dibner et al., 2007). Low bone strength increases the risk of fractures during rearing, catching, transport, unloading, and stunning, which can contribute to higher mortality, culling, and carcass condemnations (Onyango et al., 2003; Sun et al., 2018).

At TW 1, TBS was not affected by category while at TW 2, CONV birds were similar to FAST birds, yet greater than MOD and SLOW birds. When the values were expressed relative to the BW, at TW 1, CONV birds had higher TBS than the other categories, while at TW 2, CONV birds exhibited similar relative TBS compared to SLOW birds, yet greater than FAST and MOD birds. These findings are line with the results of McDevitt et al. (2006), who reported similar absolute bone breaking strength between FG and SG birds evaluated at a similar BW, indicating that bones from FG birds were as strong as or stronger than those of SG birds at the same TW. The greater relative TBS values found in CONV compared to MOD and FAST birds may be a result of more balanced selection criteria practiced by breeding companies in the past 25 yr for FG birds, which has incorporated not only growth performance traits but also skeletal integrity (Whitehead, 2007; Kapell et al., 2012; Neeteson-van Nieuwenhoven et al., 2013).

The greater relative TBS values observed in SLOW birds compared to MOD and FAST birds is likely due to the improved bone strength and quality associated with slower growth as demonstrated in other studies (Williams et al., 2004; Shim et al., 2012b), since the SG birds evaluated in our study encompassed a wide range of growth rates, with FAST and MOD birds classified as SG birds but showing ADG greater than those observed in SLOW birds. A more plausible explanation for the greater relative TBS of SLOW birds is the difference in BW observed among the categories, rather than differences in TBS per se. Despite the original plan to process the birds at a similar BW, SLOW birds did not reach the same BW as the other categories at TW 2 due to their slower growth rate. Thus, the comparisons between SLOW birds and other categories at both TWs may not accurately represent the differences at the same BW.

The age of the birds affects its bone strength, as the BW and absolute bone mass increase as the birds grow, with the latter being proportional to bone strength (Frost, 1997). The changes in bone strength as the birds age may be a result of modifications in collagen crosslink content, making the bones tougher and less brittle (Rath et al., 2000). Because FG and SG birds were processed 2 wk apart at both TWs, age differences may have influenced the differences in relative TBS among the categories observed in our study. In fact, when evaluated at the same age, CONV birds exhibited similar relative TBS to FAST and MOD, yet lower than SLOW strains. However, when BW was not considered, CONV birds had the greatest TBS at the same age. Similarly, McDevitt et al. (2006) reported greater absolute bone breaking strength in FG compared to SG birds at the same age. This indicates that the incorporation of bone health into breeding programs has been successful at improving tibia breaking strength in FG birds, suggesting that issues related to lameness are most likely not related to tibia strength. However, because lameness is a multifactorial disorder that may be triggered by disturbances in different tissues and bones (Bradshaw et al., 2002), future studies should also investigate the bone status of other pelvic limb bones, such as femur and tarsometatarsus due to their contribution to walking ability (Paxton et al., 2013, 2014). In addition, other indicators of bone quality, such as bone mineral density and bone stiffness (Rath et al., 2000), should be studied to provide a better understanding of the differences in bone health and development among strains differing in genetic potential for growth. The assessment of bone breaking strength at earlier ages would also be relevant to investigate differences among the categories due to the rapid skeletal growth in this period and the smaller disparity in BW between FG and SG strains.

#### Tibial Dimensions

At TW 1, CONV birds had shorter and narrower tibiae than birds from the other categories. Because CONV birds were 2 wk younger than the SG strains at both TWs, the morphometric differences observed may be attributed to the difference in age, as tibial length and diameter increase as birds age (Lilburn, 1994; Talaty et al., 2009; Charuta et al., 2013). The differences in body conformation between FG and SG strains at the same BW were assessed by Weimer et al. (2020), who reported longer body and greater shank length in SG birds compared to FG birds, when these birds were 63 and 42 d, respectively, which agrees with the findings reported in our study. Interestingly, at TW 2, CONV birds still had shorter tibiae than the three categories of SG birds, while the differences in tibial diameter disappeared despite the age and BW differences between FG and SG strains. The differences among categories were still present when length and diameter were expressed relative to the BW, with SLOW birds exhibiting greater values at both TWs, while the differences among the other categories differed in each TW, suggesting age and/or BW-dependent changes.

Differences in rate of increase in tibial length and diameter throughout the life cycle of the birds (Talaty et al., 2009) may have contributed to the similar values of tibial diameter among categories, despite the differences observed in tibial length at TW 2. Because bone width plays a role in bone breaking strength (Williams et al., 2004), the increase in tibial diameter observed in CONV strains from TW 1 to TW 2 may indicate greater resistance to breaking, with less susceptibility to bone fracture. However, the increase in tibial diameter observed in FG strains may be due to the accelerated increase in BW, leading to a rapid load-induced expansion of the tibiotarsus by increasing the periosteal surface of the bone as suggested by Williams et al. (2004) and Rawlinson et al. (2009). Nonetheless, this rapid increase in tibial diameter is likely not accompanied by adequate osteonal infilling by osteoblast, resulting in a more porous cortical bone (Williams et al., 2004; Rawlinson et al., 2009). Further research is needed to investigate differences in bone mineral density between FG and SG birds to determine if the enlargement in tibial diameter observed in CONV birds in this present study is accompanied by an increase in porosity of their cortical bone compared to SG birds at different ages.

At a similar age (48 d) CONV birds exhibited longer and wider tibiae, but when the values were expressed as a ratio to the BW, CONV birds were lower than the other categories, suggesting that the rapid increase in BW is not accompanied by an equally fast increase in bone size, which could lead to excessive weight for the immature bones to support (Rath, 2000). Differences in rates of increase between BW and tibial dimensions over a production cycle may have contributed to the differences observed among categories (Biesiada-Drzazga et al., 2012). Indeed, Applegate and Lilburn (2002) studied a FG strain of broiler chicken from hatch to slaughter and reported a 3.7 to 5.0-fold increase in bone morphometric traits (length and diameter), whereas a 40-fold increase in BW was observed in the same period. However, because Applegate and Lilburn (2002) did not study a SG strain, it is unknown how bone growth changes over time for SG birds and more importantly, if these changes are associated with differences in walking ability.

Even though bone characteristics (e.g., mineral content, breaking strength, mineral density, and morphology) are commonly used to assess bone quality and development, the effects of these bone characteristics on leg health and walking ability are unclear in broiler chickens. While some studies found a link between several bone traits and leg disorders or walking ability (Cruickshank and Sim, 1986; Tablante et al., 2003; Guo et al., 2019), others studies failed to confirm such relationship (Brickett et al., 2007; Talaty et al., 2010; Toscano et al., 2013).

The shorter legs of CONV birds combined with their greater breast muscle yield (Santos et al., 2021) may affect the locomotor ability (Santos et al., 2022). In fact, the increase in breast muscle yield observed in FG strains has been shown to displace their center of mass cranially (Corr et al., 2003; Paxton et al., 2014). Maintaining shorter legs may help birds control lateral motion of the center of mass and stabilize balance (Bauby and Kuo, 2000; Paxton et al., 2014). However, shorter limbs may be less efficient (Steudel-Numbers and Tilkens, 2004), which may lead to a decrease in walking and activity as a means to compensate for this greater energetic demand (Paxton et al., 2014). Although the changes in body conformation mentioned above may be associated with a decrease in activity, differences in activity can also affect bone traits. The concept that exercise and locomotor activity positively impact bone health has been widely documented in several species, including poultry (Reiter and Bessei, 1998; Kohrt et al., 2004; Pedersen et al., 2020; Pufall et al., 2021). In fact, stress and strain resulted from muscle forces and external loads are known to modulate bone remodeling, improving its mechanical function, with walking being the most common activity involved in mechanical loading of the appendicular skeleton (Shipov et al., 2010; Ruiz-Feria et al., 2014).

Recent work by Pulcini et al. (2021) found remarkable differences in tibial shape in broiler strains raised in organic systems and differing in growth rate and walking behavior, with a more pronounced curvature of the anteroposterior axis of the tibia being correlated with more static behavior (e.g., resting, roosting). A companion study evaluating the behavior, inactivity, and enrichment use of the same strains presented in this paper, revealed that CONV birds spent more time sitting, and less time standing and walking than the other SG categories at d 26 (Dawson et al., 2021). Overall, a similar pattern was also observed at d 42, with increased growth rate being associated with shorter time spent standing and walking (Dawson et al., 2021). In addition, at TW 2, an increase in growth rate was associated with a lower proportion of birds using all the enrichments provided as well as accessing the elevated platforms (Dawson et al., 2021). Therefore, the possible relationship between bone traits and behaviour among categories should not be discarded.

#### Bone Dry Matter, Mineral, and Organic Content

Tibial dry matter, ash, and organic content were only measured at TW 2, when FG and SG birds were 48 and 62 d, respectively. The determination of ash content is used as an indicator of bone mineralization. In our study, the tibiae were selected due to their essential role for BW support and because disturbances in the tibia growth plate can be associated with TD and lameness (Julian, 1998). The absolute weights of tibial traits provide a quantitative assessment of bone development, as bone mass increases during growth (Iwaniec and Turner, 2016). On the other hand, the relative values provide an “index” and quantitative indicator of bone growth in comparison to the increase in BW; this value has been shown to be altered in some bone disorders such as valgus-varus deformity (Guo et al., 2019).

Bones from the CONV birds had lighter dry matter, ash, and organic weight in comparison to FAST and MOD birds, despite the similarities in BW among these categories at TW 2. This difference in absolute weight of tibial components is likely attributed to the shorter tibial length of CONV birds at TW 2. As previously mentioned, CONV birds were 2 wk younger than the SG strains at the same TW, which may have contributed to shorter tibial length in the former (Yalcin et al., 2001; Talaty et al., 2009).

In addition, CONV birds had the lowest dry matter and ash weight relative to the BW, which may indicate that bone growth and development may not keep pace with the sharp increase in body mass (Rath et al., 2000). These findings are corroborated by McDevitt et al. (2006), who reported that at a similar BW, FG birds had shorter and lighter tibiae than SG birds. However, in the same study FG birds exhibited greater ash and lower organic content (relative to the dry matter) than SG birds. In our study, despite the differences in absolute dry matter, ash, and organic matter, the percentage of ash, organic matter, and the ratio of organic to inorganic matter did not differ among categories. In addition, CONV birds had similar ash per unit of length of the tibia compared to the other categories. Similar results were reported by Talaty et al. (2009), who did not find differences in bone mineral content (determined through dual-energy x-ray) among different commercial strains of broiler chickens at different ages. However, all the strains evaluated had similar BW and growth rate, which differs from our study.

Differences in bone morphology and mineralization between 2 commercial strains of broiler chickens were reported by Yalcin et al. (2001) during the first 16 d of age, whereas at later ages these differences disappeared. Similarly, Shim et al. (2012b) reported no difference in ash content between FG and SG strains evaluated at 6 wk of age. It is important to emphasize that in the present study bone dry matter, mineral, and organic content were only evaluated at one time point, at TW 2, when birds were expected to be about 3.2 kg. However, due to the changes in bone traits over a production cycle, more research into bone mineral content and density at different time points is warranted to determine if bone development and susceptibility to leg disorders differ among strains at earlier stages of growth, due to the rapid increase in BW and bone growth in this period.

Overall tibial ash content (%) and tibial morphometric traits and breaking strength (absolute and relative to the BW) reported in our study for FG and SG strains at both TWs are within the normal range found in sound birds (based on gait score assessment) at slaughter age (34–40 d) reported by Alkhtib et al. (2021). However, because Alkhtib et al. (2021) only evaluated a FG strain of broiler chicken, the normal range of the aforementioned variables for strains differing in genetic potential for growth remains to be elucidated.

#### Tibial Dyschondroplasia

In our study, TD was mainly found in MOD and FAST birds. This suggests that genetic selection for leg health variables has not been as intense in SG birds as in FG birds, likely due to their reduced growth performance that is associated with lower incidence of leg disorders. However, because birds showing signs of lameness were promptly euthanized in our study, the incidence of TD may be higher than the values reported, although the overall mortality and cull rates were low (Torrey et al., 2021). Although the presence of TD has been documented to negatively affect bird's walking ability based on gait score assessment (Sanotra et al., 2001), this finding has not been supported by other researchers (Fernandes et al., 2012). Because the lesions observed in our study were mild (presence of irregular cartilage in less than one third of the growth plate) (Edwards and Veltmann, 1983; Sanotra et al., 2001), the walking ability of birds exhibiting TD lesions was likely not affected by the condition.

## CONCLUSIONS

Based on the results of this study, differences in growth rate were associated with differences in most of the bone traits examined at a similar TW and at a similar age. Morphometric traits differed by category, with CONV birds having shorter absolute tibial length at both TWs and the shortest tibial length relative to the BW at TW 2 and at 48 d of age. However, both absolute TBS and TBS relative to BW of CONV were similar or greater than SG birds at both TWs. Nonetheless, at a similar age, CONV birds had the greatest TBS, yet lower than SLOW when differences were adjusted for BW. Ash content did not differ among categories at TW 2, suggesting similar bone mineralization among categories. These results suggest that differences in functional abilities of CONV compared to SG birds at a similar BW may be due to morphometric differences rather than differences in bone strength and bone mineralization. Other bone quality indicators (e.g., bone stiffness, bone mineral density, chemical composition, and cortical thickness) would be useful to provide a better understanding of bone development at different stages of growth over a life cycle of FG and SG birds especially at earlier stages that are characterized by a rapid increase in body mass and bone development.

This research was funded by the Global Animal Partnership, the Canada First Research Excellence Fund (through Food from Thought), and Ontario Agri-Food Innovation Alliance.

## Appendix Supplementary materials

Image, application 1

## Footnotes

Supplementary material associated with this article can be found, in the online version, at doi:10.1016/j.psj. 2022.101755.
