# Supplementary material for: In pursuit of a better broiler: tibial morphology, breaking strength, and ash content in conventional and slower-growing strains of broiler chickens
Source: Poult Sci. 2022 Jan 30;101(4):101755. doi: 10.1016/j.psj.2022.101755 (PMC8914365; doi:10.1016/j.psj.2022.101755)
Supplement: Supplementary file 1 [file mmc1.docx]

**Table 1:** Differences in body weight (BW), tibial breaking strength (TBS), tibial morphology, and tibial ash and organic content (LS-means ± SEM) among CONV strains at Target Weights 1 and 2. At Target Weights 1 and 2, CONV strains were 34 and 48 days, respectively.

|  | **Strain** | | |
| --- | --- | --- | --- |
| **Variable** | **B** | | **C** |
| **Target Weight 1^1^** |  | |  |
| BW (g) | 1,953 ± 56.5 | 1,766 ± 58.8 | |
| TBS (N)^2^ | 326.5 ± 18.67 | 257.6 ± 16.73 | |
| TBS:BW (N/kg)^3^ | 167.2. ± 9.25 | 146.0 ± 9.04 | |
| Diameter (mm) | 7.47 ± 0.196 | 6.95 ± 0.211 | |
| Length (mm) | 98.2 ± 1.01^a^ | 93.1 ± 1.09^b^ | |
| Diameter:BW (mm/kg) | 3.80 ± 0.104 | 3.94 ± 0.123 | |
| Length:BW (mm/kg) | 50.00 ± 1.224 | 52.70 ± 1.473 | |
| Length:Diameter | 13.14 ± 0.294 | 13.42 ± 0.345 | |
| **Target Weight 2^4^** |  |  | |
| BW (g) | 3,298 ± 89.7 | 3,231 ± 79.9 | |
| TBS (N) | 376.6 ± 19.81 | 349.5± 15.95 | |
| TBS:BW (N/kg) | 114.2 ± 5.81 | 108.1 ± 4.81 | |
| Diameter (mm) | 9.29 ± 0.223 | 8.82 ± 0.183 | |
| Length (mm) | 118.6 ± 1.09 | 115.4 ± 0.99 | |
| Diameter:BW (mm/kg) | 2.81 ± 0.069 | 2.74 ± 0.059 | |
| Length:BW (mm/kg) | 35.95 ± 0.798 | 35.70 ± 0.693 | |
| Length:Diameter | 12.75 ± 0.257 | 13.08 ± 0.232 | |
| Dry matter wt (g)^5^ | 10.12 ± 0.305 | 9.84 ± 0.285 | |
| Ash wt (g) | 4.06 ± 0.134 | 3.81 ± 0.125 | |
| Organic matter wt (g) | 6.06 ± 0.183 | 6.02 ± 0.174 | |
| Dry matter wt:BW (%) | 3.05 ± 0.063 | 3.04 ± 0.057 | |
| Ash wt:BW (%) | 1.22 ± 0.022 | 1.17 ± 0.021 | |
| Ash content (%) | 40.24 ± 0.540 | 38.60 ± 0.489 | |
| Organic matter (%) | 59.76 ± 0.540 | 61.40 ± 0.489 | |
| Organic:Inorganic | 1.49 ± 0.035 | 1.59 ± 0.032 | |
| Ash:Length (g/mm) | 0.34 ± 0.009 | 0.34 ± 0.009 | |

^1^ Number of birds per strain at Target Weight 1: B: n = 19; C: n = 15.

^2^ Absolute tibial breaking strength (TBS). Maximum TBS expressed in newtons (N).

^3^ Relative tibial breaking strength (TBS). Maximum TBS was obtained in newtons (N) and adjusted for the BW.

^4^ Number of birds per strain at Target Weight 2: B: n = 23; C: n =31.

^5^ Tibial dry matter, and organic and inorganic content were only obtained at Target Weight 2.

^a-b^ Different superscripts within the same row represent significant differences between categories (P < 0.05).

**Table 2:** Differences in body weight (BW), tibial breaking strength (TBS), tibial morphology, and tibial ash and organic content (LS-means ± SEM) among FAST strains at Target Weights 1 and 2. At Target Weights 1 and 2, FAST strains were 48 and 62 days, respectively.

|  | **Strain** | | | |
| --- | --- | --- | --- | --- |
| **Variable** | **F** | **G** | **I** | **M** |
| **Target Weight 1^1^** |  |  |  |  |
| BW (g) | 2,617 ± 87.2 | 2,470 ± 68.0 | 2,428 ± 66.0 | 2,567 ± 86.9 |
| TBS (N)^2^ | 303.5 ± 19.72 | 319.5 ± 17.10 | 286.5 ± 15.37 | 292.8 ± 18.54 |
| TBS:BW (N/kg)^3^ | 116.0 ± 7.29 | 129.0 ± 6.65 | 118.0 ± 6.17 | 114.1 ± 7.02 |
| Diameter (mm) | 9.24 ± 0.271 | 8.46 ± 0.207 | 8.58 ± 0.206 | 8.12 ± 0.238 |
| Length (mm) | 113.8 ± 1.28 | 116.2 ± 1.09 | 115.7 ± 1.06 | 118.7 ± 1.40 |
| Diameter:BW (mm/kg) | 3.53 ± 0.106 | 3.41 ± 0.086 | 3.54 ± 0.087 | 3.16 ± 0.095 |
| Length:BW (mm/kg) | 43.48 ± 1.182 | 47.04 ± 1.065 | 47.66 ± 1.059 | 46.00 ± 1.324 |
| Length:Diameter | 12.31 ± 0.304^b^ | 13.72 ± 0.284^a^ | 13.48 ± 0.271^ab^ | 14.62 ± 0.371^a^ |
| **Target Weight 2^4^** |  |  |  |  |
| BW (g) | 3,497 ± 84.2 | 3,419 ± 92.9 | 3,467 ± 95.3 | 3,366 ± 11.2 |
| TBS (N) | 327.4 ± 15.34 | 321.5 ± 16.62 | 317.2 ± 17.77 | 397.6 ± 25.17 |
| TBS:BW (N/kg) | 93.6 ± 4.16 | 94.0 ± 4.67 | 91.5 ± 4.94 | 118.1 ± 7.22 |
| Diameter (mm) | 9.61 ± 0.203 | 9.13 ± 0.227 | 9.48 ± 0.232 | 9.03 ± 0.269 |
| Length (mm) | 126.6 ± 1.07^b^ | 129.4 ± 1.25^ab^ | 133.3 ± 1.24^a^ | 130.6 ± 1.50^ab^ |
| Diameter:BW (mm/kg) | 2.75 ± 0.059 | 2.70 ± 0.071 | 2.72 ± 0.069 | 2.68 ± 0.083 |
| Length:BW (mm/kg) | 36.20 ± 0.706 | 37.80 ± 0.897 | 38.42 ± 0.863 | 38.80 ± 1.072 |
| Length:Diameter | 13.15 ± 0.238 | 14.15 ± 0.298 | 14.05 ± 0.291 | 14.47 ± 0.364 |
| Dry matter wt (g)^5^ | 11.30 ± 0.266 | 11.50 ± 0.298 | 11.56 ± 0.298 | 12.15 ± 0.360 |
| Ash wt (g) | 4.29 ± 0.129 | 4.61 ± 0.143 | 4.79 ± 0.144 | 4.78 ± 0.173 |
| Organic matter wt (g) | 7.01 ± 0.158 | 6.89 ± 0.176 | 6.77 ± 0.176 | 7.37 ± 0.212 |
| Dry matter wt:BW (%) | 3.23 ± 0.061^b^ | 3.36 ± 0.070^ab^ | 3.30 ± 0.070^ab^ | 3.60 ± 0.084^a^ |
| Ash wt:BW (%) | 1.22 ± 0.025^b^ | 1.34 ± 0.028^ab^ | 1.38 ± 0.028^a^ | 1.41 ± 0.033^a^ |
| Ash content (%) | 38.15 ± 0.479^b^ | 39.81 ± 0.541^ab^ | 41.39 ± 0.541^a^ | 39.14 ± 0.650^ab^ |
| Organic matter (%) | 61.85 ± 0.497^a^ | 60.18 ± 0.541^ab^ | 58.61 ± 0.541^b^ | 60.86 ± 0.650^ab^ |
| Organic:Inorganic | 1.63 ± 0.031^a^ | 1.52 ± 0.035^ab^ | 1.42 ± 0.035^b^ | 1.57 ± 0.041^ab^ |
| Ash:Length (g/mm) | 0.34 ± 0.009 | 0.34 ± 0.009 | 0.36 ± 0.009 | 0.36 ± 0.011 |

^1^ Number of birds per strain at Target Weight 1: F: n = 16; G: n = 22; I: n = 24; M: n = 16.

^2^ Absolute tibial breaking strength (TBS). Maximum TBS expressed in newtons (N).

^3^ Relative tibial breaking strength (TBS). Maximum TBS was obtained in newtons (N) and adjusted for the BW.

^4^ Number of birds per strain at Target Weight 2: F: n = 32; G: n = 24; I: n = 23; M: n = 16.

^5^ Tibial dry matter, and organic and inorganic content were only obtained at Target Weight 2.

^a-b^ Different superscripts within the same row represent significant differences between categories (P < 0.05).

**Table 3:** Differences in body weight (BW), tibial breaking strength (TBS), tibial morphology, and tibial ash and organic content (LS-means ± SEM) among MOD strains at Target Weights 1 and 2. At Target Weights 1 and 2, MOD strains were 48 and 62 days, respectively.

|  |  | **Strain** | | | |
| --- | --- | --- | --- | --- | --- |
| **Variable** | | **E** | **H** | **O** | **S** |
| **Target Weight 1^1^** | |  |  |  |  |
| BW (g) | | 2,551 ± 70.1 | 2,226 ± 74.1 | 2,434 ± 66.2 | 2,241 ± 61.6 |
| TBS (N)^2^ | | 308.3 ± 16.21 | 249.8 ± 16.24 | 277.7 ± 14.60 | 304.9 ± 16.03 |
| TBS:BW (N/kg)^3^ | | 120.8 ± 6.14 | 112.2 ± 7.07 | 114.1 ± 5.79 | 136.2 ± 6.91 |
| Diameter (mm) | | 8.64 ± 0.209 | 7.75 ± 0.235 | 8.61 ± 0.206 | 8.33 ± 0.202 |
| Length (mm) | | 114.9 ± 1.07 | 109.6 ± 1.29 | 113.5 ± 1.04 | 111.9 ± 1.04 |
| Diameter:BW (mm/kg) | | 3.38 ± 0.080 | 3.50 ± 0.111 | 3.54 ± 0.087 | 3.72 ± 0.093 |
| Length:BW (mm/kg) | | 45.02 ± 1.012 | 49.30 ± 1.401 | 46.63 ± 1.035 | 49.93 ± 1.122 |
| Length:Diameter | | 13.29 ± 0.271 | 14.12 ± 0.363 | 13.17 ± 0.265 | 13.42 ± 0.274 |
| **Target Weight 2^4^** | |  |  |  |  |
| BW (g) | | 3,198 ± 87.9 | 3,101 ± 74.1 | 3,368 ± 91.6 | 3,080 ± 83.7 |
| TBS (N) | | 339.3 ± 17.84 | 338.8 ± 15.25 | 282.4 ± 15.11 | 317.6 ± 16.69 |
| TBS:BW (N/kg) | | 106.0 ± 5.38^ab^ | 109.1 ± 4.71^a^ | 83.9 ± 4.34^b^ | 103.1 ± 5.24^ab^ |
| Diameter (mm) | | 9.55 ± 0.236 | 8.99 ± 0.189 | 9.48 ± 0.227 | 9.15 ± 0.221 |
| Length (mm) | | 131.2 ± 1.26 | 126.2 ± 1.04 | 129.8 ± 1.19 | 126.2 ± 1.17 |
| Diameter:BW (mm/kg) | | 2.97 ± 0.076 | 2.88 ± 0.062 | 2.81 ± 0.069 | 2.97 ± 0.074 |
| Length:BW (mm/kg) | | 41.00 ± 0.940 | 40.70 ± 0.792 | 38.51 ± 0.855 | 40.98 ± 0.921 |
| Length:Diameter | | 13.74 ± 0.288 | 14.05 ± 0.249 | 13.70 ± 0.276 | 13.78 ± 0.282 |
| Dry matter wt (g)^5^ | | 11.40 ± 0.329 | 10.77 ± 0.297 | 10.93 ± 0.329 | 10.29 ± 0.329 |
| Ash wt (g) | | 4.63 ± 0.149 | 4.25 ± 0.134 | 4.25 ± 0.149 | 4.08 ± 0.149 |
| Organic matter wt (g) | | 6.77 ± 0.209 | 6.53 ± 0.189 | 6.68 ± 0.209 | 6.20 ± 0.209 |
| Dry matter wt:BW (%) | | 3.56 ± 0.088 | 3.47 ± 0.078 | 3.24 ± 0.088 | 3.34 ± 0.088 |
| Ash wt:BW (%) | | 1.44 ± 0.037^a^ | 1.37 ± 0.034^ab^ | 1.25 ± 0.037^b^ | 1.32 ± 0.036^ab^ |
| Ash content (%) | | 40.55 ± 0.669 | 39.40 ± 0.592 | 38.86 ± 0.669 | 39.70 ± 0.669 |
| Organic matter (%) | | 59.44 ± 0.670 | 60.59 ± 0.592 | 61.13 ± 0.670 | 60.30 ± 0.670 |
| Organic:Inorganic | | 1.47 ± 0.047 | 1.55 ± 0.042 | 1.62 ± 0.047 | 1.53 ± 0.047 |
| Ash:Length (g/mm) | | 0.35 ± 0.010 | 0.33 ± 0.009 | 0.32 ± 0.009 | 0.32 ± 0.009 |

^1^ Number of birds per strain at Target Weight 1: E: n = 23; H: n = 15; O: n = 24; S: n = 23.

^2^ Absolute tibial breaking strength (TBS). Maximum TBS expressed in newtons (N).

^3^ Relative tibial breaking strength (TBS). Maximum TBS was obtained in newtons (N) and adjusted for the BW.

^4^ Number of birds per strain at Target Weight 2: E: n = 23; H: n = 32; O: n = 23; S: n = 23.

^5^ Tibial dry matter, and organic and inorganic content were only obtained at Target Weight 2.

^a-b^ Different superscripts within the same row represent significant differences between categories (P < 0.05).

**Table 4:** Differences in body weight (BW), tibial breaking strength (TBS), tibial morphology, and tibial ash and organic content (LS-means ± SEM) among SLOW strains at Target Weights 1 and 2. At Target Weights 1 and 2, SLOW strains were 48 and 62 days, respectively.

|  | **Strain** | | | |  |
| --- | --- | --- | --- | --- | --- |
| **Variable** | **D** | **J** | **K** | **N** |  |
| **Target Weight 1^1^** |  |  |  |  |  |
| BW (g) | 1,993 ± 66.3 | 2,132 ± 57.9 | 1,967 ± 53.5 | 1,970 ± 53.6 |  |
| TBS (N)^2^ | 273.9 ± 17.34 | 265.5 ± 13.96 | 269.6 ± 14.18 | 287.8 ± 15.13 |  |
| TBS:BW (N/kg)^3^ | 137.5 ± 8.41 | 124.4 ± 6.32 | 137.1 ± 6.88 | 146.0 ± 7.40 |  |
| Diameter (mm) | 7.58 ± 0.226 | 8.59 ± 0.206 | 8.13 ± 0.197 | 8.46 ± 0.205 |  |
| Length (mm) | 110.9 ± 1.27 | 114.3 ± 1.05 | 110.9 ± 1.03 | 110.9 ± 1.03 |  |
| Diameter:BW (mm/kg) | 3.80 ± 0.116 | 4.03 ± 0.099 | 4.10 ± 0.102 | 4.29 ± 0.107 |  |
| Length:BW (mm/kg) | 55.61 ± 1.526 | 53.61 ± 1.191 | 56.40 ± 1.254 | 56.19 ± 1.263 |  |
| Length:Diameter | 14.63 ± 0.368^a^ | 13.30 ± 0.268^ab^ | 13.65 ± 0.278^ab^ | 13.10 ± 0.267^b^ |  |
| **Target Weight 2^4^** |  |  |  |  |  |
| BW (g) | 2,838 ± 68.1 | 3,022 ± 81.5 | 2,764 ± 75.1 | 2,760 ± 75.9 |  |
| TBS (N) | 377.6 ± 16.99 | 308.8 ± 15.73 | 300.6 ± 15.54 | 301.7 ± 16.19 |  |
| TBS:BW (N/kg) | 133.0 ± 5.77^a^ | 102.2 ± 5.04^b^ | 108.8 ± 5.43^ab^ | 109.3 ± 5.66^ab^ |  |
| Diameter (mm) | 8.89 ± 0.186 | 9.57 ± 0.230 | 9.04 ± 0.217 | 9.57 ± 0.236 |  |
| Length (mm) | 127.3 ± 1.07 | 129.9 ± 1.19 | 126.4 ± 1.16 | 127.0 ± 1.21 |  |
| Diameter:BW (mm/kg) | 3.12 ± 0.067 | 3.17 ± 0.078 | 3.27 ± 0.081 | 3.46 ± 0.088 |  |
| Length:BW (mm/kg) | 44.88 ± 0.883 | 43.00 ± 0.958 | 45.72 ± 1.015 | 46.04 ± 1.052 |  |
| Length:Diameter | 14.30 ± 0.255 | 13.58 ± 0.275 | 13.98 ± 0.282 | 13.28 ± 0.276 |  |
| Dry matter wt (g)^5^ | 10.96 ± 0.299 | 10.54 ± 0.328 | 10.33 ± 0.332 | 9.73 ± 0.344 |  |
| Ash wt (g) | 4.26 ± 0.126 | 4.32 ± 0.139 | 4.08 ± 0.141 | 4.07 ± 0.145 |  |
| Organic matter wt (g) | 6.69 ± 0.194^a^ | 6.22 ± 0.211^ab^ | 6.24 ± 0.215^ab^ | 5.66 ± 0.223^b^ |  |
| Dry matter wt:BW (%) | 3.86 ± 0.076 | 3.48 ± 0.085 | 3.70 ± 0.086 | 3.50 ± 0.090 |  |
| Ash wt:BW (%) | 1.50 ± 0.031 | 1.42 ± 0.034 | 1.47 ± 0.034 | 1.47 ± 0.036 |  |
| Ash content (%) | 39.21 ± 0.584 | 40.89 ± 0.647 | 39.60 ± 0.659 | 41.70 ± 0.687 |  |
| Organic matter (%) | 60.79 ± 0.584 | 59.10 ± 0.647 | 60.40 ± 0.659 | 58.30 ± 0.687 |  |
| Organic:Inorganic | 1.56 ± 0.035 | 1.46 ± 0.038 | 1.54 ± 0.039 | 1.41 ± 0.041 |  |
| Ash:Length (g/mm) | 0.33 ± 0.009 | 0.33 ± 0.009 | 0.32 ± 0.009 | 0.32 ± 0.010 | |

^1^ Number of birds per strain at Target Weight 1: D: n = 16; J: n = 24; K: n = 23; N: n = 23.

^2^ Absolute tibial breaking strength (TBS). Maximum TBS expressed in newtons (N).

^3^ Relative tibial breaking strength (TBS). Maximum TBS was obtained in newtons (N) and adjusted for the BW.

^4^ Number of birds per strain at Target Weight 2: D: n = 32; J: n = 25; K: n = 24; N: n = 22.

^5^ Tibial dry matter, and organic and inorganic content were only obtained at Target Weight 2.

^a-b^ Different superscripts within the same row represent significant differences between categories (P < 0.05).

**Table 5: Effect of category and sex on tibial traits (LS-means ± SEM) at Target weight 1 and 2.** At Target Weight 1, conventional and slower strains birds were 34 and 48 d of age, respectively. At Target Weight 2, conventional and the remaining categories were 48 and 62 days, respectively.

|  |  | **Category** | | | |
| --- | --- | --- | --- | --- | --- |
| **Variable** | **Sex** | **CONV** | **FAST** | **MOD** | **SLOW** |
| **Target Weight 1** |  |  |  |  |  |
| Length (mm) | F | 93.8 ± 0.89^c^ | 112.9 ± 0.73^az^ | 109.9 ± 0.69^bz^ | 108.8 ± 0.68^bz^ |
|  | M | 97.5 ± 1.05^c^ | 119.4 ± 0.81^ay^ | 115.1 ± 0.72^by^ | 114.88 ± 0.72^by^ |
| **Target Weight 2^1^** |  |  |  |  |  |
| Length (mm) | F | 113.5 ± 1.05^bz^ | 125.1 ± 0.83^az^ | 123.3 ± 0.78^az^ | 123.2 ± 0.77^az^ |
|  | M | 120.6 ± 0.88^by^ | 135.0 ± 0.81^ay^ | 133.4 ± 0.74^ay^ | 132.2 ± 0.74^ay^ |
| Dry wt (g) | F | 8.78 ± 0.294^z^ | 9.60 ± 0.203^z^ | 9.02 ± 0.222^z^ | 8.87 ± 0.220^z^ |
|  | M | 11.17 ± 0.243^cy^ | 13.64 ± 0.183^ay^ | 12.67 ± 0.198^by^ | 11.90 ± 0.205^bcy^ |
| Ash wt (g) | F | 3.45 ± 0.128^z^ | 3.76 ± 0.099^z^ | 3.54 ± 0.101^z^ | 3.52 ± 0.091^z^ |
|  | M | 4.41 ± 0.106^cy^ | 5.47 ± 0.089^ay^ | 5.06 ± 0.089^by^ | 4.85 ± 0.085^by^ |
| Organic matter (g) | F | 5.31 ± 0.185^z^ | 5.85 ± 0.125^z^ | 5.48 ± 0.147^z^ | 5.34 ± 0.148^z^ |
|  | M | 6.77 ± 0.511^cy^ | 8.17 ± 0.111^ay^ | 7.61 ± 0.129^by^ | 7.07 ± 0.137^bcy^ |
| Ash:BW (g/kg) | F | 1.16 ± 0.023^b^ | 1.23 ± 0.021^bz^ | 1.25 ± 0.027^bz^ | 1.37 ± 0.025^az^ |
|  | M | 1.23 ± 0.018^c^ | 1.41 ± 0.019^by^ | 1.41 ± 0.024^by^ | 1.53 ± 0.023^ay^ |
| Ash:Length (g/mm) | F | 0.31 ± 0.009^z^ | 0.30 ± 0.006^z^ | 0.29 ± 0.007^z^ | 0.29 ± 0.007^z^ |
|  | M | 0.36 ± 0.007^by^ | 0.40 ± 0.006^ay^ | 0.38 ± 0.006^aby^ | 0.36 ± 0.006^by^ |

^1^ Tibial dry matter, organic, and inorganic content were only obtained at Target Weight 2.

^a-c^ Different superscripts within the same row represent significant differences between categories for each sex (P< 0.05).

^y-z^ Different superscripts within the same column for the same parameter represent significant differences between sexes in each category (P < 0.05).

**Table 6: Body weight (BW), tibial breaking strength (TBS), tibial morphology, and tibial ash and organic content at Target Weights 1 and 2**. Only descriptive data included (raw means ± standard deviation).

|  | **Strain^1^** | |
| --- | --- | --- |
| **Variable** | **A** | **T** |
| **Target Weight 1^2^** |  |  |
| BW (g) | 1,652 ± 149.4 | N/A |
| TBS (N)^3^ | 313.3 ± 71.60 | N/A |
| TBS:BW (N/kg)^4^ | 188.2 ± 31.19 | N/A |
| Diameter (mm) | 7.41 ± 0.864 | N/A |
| Length (mm) | 91.88 ± 4.320 | N/A |
| Diameter:BW (mm/kg) | 4.49 ± 0.409 | N/A |
| Length:BW (mm/kg) | 55.87 ± 4.031 | N/A |
| Length:Diameter | 12.50 ± 1.309 | N/A |
| **Target Weight 2^5^** |  |  |
| BW (g) | 3,070 ± 414.7 | 1,019 ± 101.7 |
| TBS (N) | 371.9 ± 88.18 | 185.3 ± 40.41 |
| TBS:BW (N/kg) | 120.9 ± 21.32 | 183.4 ± 42.80 |
| Diameter (mm) | 9.00 ± 1.098 | 6.78 ± 0.436 |
| Length (mm) | 118.1 ± 4.71 | 104.9 ± 5.01 |
| Diameter:BW (mm/kg) | 2.95 ± 0.235 | 6.75 ± 0.452 |
| Length:BW (mm/kg) | 39.14 ± 5.238 | 104.7 ± 7.12 |
| Length:Diameter | 13.23 ± 1.236 | 15.51 ± 0.777 |
| Dry matter wt (g)^6^ | 9.96 ± 1.774 | 4.87 ± 0.874 |
| Ash wt (g) | 4.13 ± 0.758 | 1.79 ± 0.313 |
| Organic matter wt (g) | 5.83 ± 1.062 | 3.07 ± 0.582 |
| Dry matter wt:BW (%) | 3.23 ± 0.228 | 4.85 ± 0.788 |
| Ash wt:BW (%) | 1.34 ± 0.117 | 1.79 ± 0.304 |
| Ash content (%) | 41.50 ± 1.869 | 36.98 ± 1.962 |
| Organic matter (%) | 58.49 ± 1.868 | 63.01 ± 1.962 |
| Organic:Inorganic | 1.41 ± 0.109 | 1.71 ± 0.142 |
| Ash: Length (g/mm) | 0.347 ± 0.056 | 0.171 ± 0.026 |

Due to the reduced sample size, only descriptive statistics are provided for strains A (Fast growing; ADG _0-48_= 62.65 g/d) and T (Slower growing; ADG _0-62_= 19.78 g/d). Because strain T had the lowest ADG among the strains, leg traits were only obtained at TW 2.

^2^ Number of birds per strain at Target Weight 1: A: n= 6.

^3^ Absolute tibial breaking strength (TBS). Maximum TBS expressed in newtons (N).

^4^ Relative tibial breaking strength (TBS). Maximum TBS was obtained in newtons (N) and adjusted for the BW.

^5^ Number of birds per strain at Target Weight 2: A: n= 7; T: n=11.

^6^ Tibial dry matter, and organic, and inorganic content were only obtained at Target Weight 2.

**Tibial breaking strength – Comparison of loading rate**

It is worth mentioning that the methodology used in our study to determine tibia breaking strength differed from previous studies regarding freezing procedures and loading rate. Due to logistics of the project, the samples were frozen and thawed before the determination of breaking strength. Previous studies have demonstrated that bone strength (Wilson et al., 1990) and ash content (Park et al., 2003) are not affected by freezing and thawing. In addition, because all the bones samples were submitted to the same procedure before analyses were conducted, the results were likely not affected by the methods used in our study. Although the loading rate used in our study (20 mm/s) has been previously reported (Rath et al., 1999), more recent studies conducted in poultry species have adopted a much slower loading rate, commonly ranging from 5*-*50 mm/min (Park et al., 2003; Whitehead et al., 2004; Shim et al., 2012; Candelotto et al., 2020). In fact, Crenshaw et al. (1981) reported that differences in procedures used to analyze breaking strength in swine led to variation in values found for bone breaking strength. The authors suggested that a loading speed of 5 mm/min should be used to evaluate bone breaking strength when a 3-point bending test is used due to the effects of loading rate on bone mechanical properties. Similarly, a loading speed at a rate of 30*-*60 mm/min has been suggested for evaluating bone breaking strength in mice (Jepsen et al., 2015). It was also emphasized that a lower loading speed rate ranging from 3-6 mm/min may be more accurate to detect some differences in bone mechanical properties such as post yield displacement.

In order to test if the higher loading speed adopted in our study interfered with the results found, the right tibia samples from 40 birds, representing 10 strains (4 samples per strain) were analyzed using a loading rate of 50 mm/min and compared to the left tibia of the same birds, which were analyzed using a higher loading rate of 20 mm/s. The loading speeds tested had no effect on TBS (Low speed = 369.6 ± 16.60 N; High speed =359.8 ± 16.67 N, P= 0.383), suggesting that the high loading speed used in our study did not significantly impact our results. However, future studies should use low loading speed, due to the possible impacts on bone mechanical properties and to allow comparisons with other studies that adopted a lower speed.

**References related to tibial breaking strength methodology**

Candelotto, L., M. Stadelmann, S. G. Gebhardt-Henrich, A. Stratmann, T. G. H. van de Braak, D. Guggisberg, P. Zysset, and M. J. Toscano. 2020. Genetic variation of keel and long bone skeletal properties for 5 lines of laying hens. J. Appl. Poult. Res. 29:937–946.

Crenshaw, T. D., E. R. Peo, A. J. Lewis, and B. D. Moser. 1981. Bone Strength as a Trait for Assessing Mineralization in Swine: a Critical Review of Techniques Involved. J. Anim. Sci. 53:827–835.

Jepsen, K. J., M. J. Silva, D. Vashishth, X. E. Guo, and M. C. H. Van Der Meulen. 2015. Establishing biomechanical mechanisms in mouse models: Practical guidelines for systematically evaluating phenotypic changes in the diaphyses of long bones. J. Bone Miner. Res. 30:951–966.

Park, S. Y., S. G. Birkhold, L. F. Kubena, D. J. Nisbet, and S. C. Ricke. 2003. Effect of storage condition on bone breaking strength and bone ash in laying hens at different stages in production cycles. Poult. Sci. 82:1688–1691.

Rath, N. C., J. M. Balog, W. E. Huff, G. R. Huff, G. B. Kulkarni, and J. F. Tierce. 1999. Comparative differences in the composition and biomechanical properties of tibiae of seven- and seventy-two-week-old male and female broiler breeder chickens. Poult. Sci. 78:1232–1239.

Shim, M. Y., A. B. Karnuah, A. D. Mitchell, N. B. Anthony, G. M. Pesti, and S. E. Aggrey. 2012. The effects of growth rate on leg morphology and tibia breaking strength, mineral density, mineral content, and bone ash in broilers. Poult. Sci. 91:1790–1795.

Whitehead, C. C., H. A. McCormack, L. McTeir, and R. H. Fleming. 2004. High vitamin D3 requirements in broilers for bone quality and prevention of tibial dyschondroplasia and interactions with dietary calcium, available phosphorus and vitamin A. Br. Poult. Sci. 45:425–436.

Wilson, J. H., J. P. Mason, and W. L. Beane. 1990. Influence of calcium and phosphorus on bone-strength of spent hens. Trans. Am. Soc. Agric. Eng. 33:642–647.
